# Beyond Same-Sex Attraction: Gender-Variant-Based Victimization Is Associated with Suicidal Behavior and Substance Use for Other-Sex Attracted Adolescents

**DOI:** 10.1371/journal.pone.0129976

**Published:** 2015-06-12

**Authors:** Michael Ioerger, Kimberly L. Henry, Peter Y. Chen, Konstantin P. Cigularov, Rocco G. Tomazic

**Affiliations:** 1 Department of Psychology, Syracuse University, Syracuse, New York, United State of America; 2 Department of Psychology, Colorado State University, Fort Collins, Colorado, United States of America; 3 Department of Psychology, Auburn University, Auburn, Alabama, United States of America; 4 Department of Psychology, Old Dominion University, Norfolk, Virginia, United States of America; 5 Borough of Freehold Public Schools, Freehold, New Jersey, United States of America; Emory University's RSPH, UNITED STATES

## Abstract

Gender-variant-based victimization is victimization based on the way others perceive an individual to convey masculine, feminine, and androgynous characteristics through their appearance, mannerisms, and behaviors. Previous work identifies gender-variant-based victimization as a risk factor for health-risking outcomes among same-sex attracted youths. The current study seeks to examine this relationship among other-sex attracted youths and same-sex attracted youth, and determine if gender-variant-based victimization is similarly or differentially associated with poor outcomes between these two groups. Anonymous data from a school-based survey of 2,438 racially diverse middle and high school students in the Eastern U.S. was examined. For other-sex attracted adolescents, gender-variant-based victimization was associated with a higher odds of suicidal thoughts and behaviors, regular use of cigarettes, and drug use. When compared to same-sex attracted adolescents, the harmful relationship between gender-variant-based victimization and each of these outcomes was similar in nature. These findings suggest that gender-variant-based victimization has potentially serious implications for the psychological wellbeing and substance use of other-sex attracted adolescents, not just same-sex attracted adolescents, supporting the need to address gender expression as a basis for victimization separate from sexuality- or gender-minority status. The impact that gender-variant-based victimization has on all adolescents should not be overlooked in research and interventions aimed at addressing sexual orientation-based and gender-variant-based victimization, substance use, and suicide prevention.

## Introduction

In recent years, a lot of attention has been given to the victimization that same-sex attracted middle school- and high school-aged adolescents face because of its associations with suicidal behavior and substance use [1,2]. This has resulted in researchers, educators, and policy makers emphasizing same-sex attraction as a key risk factor for being victimized and experiencing negative health outcomes [3–6]. While the attention given to same-sex attracted adolescents is warranted given the elevated risk of experiencing negative health outcomes for sexual minority adolescents [1,7], same-sex attracted adolescents have heterogeneous experiences with being victimized. Work exploring victimization among same-sex attracted adolescents has demonstrated that they may be targeted for both sexual orientation-based victimization (victimization based on individuals’ perceived or actual sexual orientation) and gender-variant-based victimization (victimization based on the way others perceive individuals to convey masculine, feminine, and androgynous characteristics through their appearance, mannerisms, and behaviors), with same-sex attracted adolescents who are targeted for gender-variant-based victimization experiencing more negative health outcomes than same-sex adolescents who are not targeted for this type of victimization [8,9].

However, in all of this, little work on gender-variant-based victimization has addressed the outcomes experienced by other-sex attracted students who also experience this type of victimization, but are not part of the sexual- or gender-minority (i.e., other-sex attracted students who are not transgender, and who do not identify as gender nonconforming). To address this limitation, the current work explores the association between gender-variant-based victimization in other-sex attracted adolescents and health-risking suicidal behaviors and substance use. Additionally, to help put the impact of this type of victimization into perspective, this work separates out the effects of attraction and gender-variant-based victimization to allow for comparisons between other-sex attracted adolescents and same-sex attracted adolescents who experience gender-variant-based victimization.

### Being Targeted for Gender-Variant-Based Victimization

Adolescents may be targeted for victimization by their peers because they are perceived to be violating gender norms. This type of victimization is a form of gender policing, which uses victimization to rebuke both male and female peers who do not conform to traditional gender norms [10,11]. In recent years, much of the work exploring the association between gender variance and negative outcomes has focused on sexual-minority adolescents because identifying as same-sex attracted is viewed as a violation of traditional gender norms [8,9]. However, any adolescents that are not perceived as expressing their gender in ways that meet prescribed social norms are potential targets for this type of victimization [11]. Indeed, research has shown that transgender and gender non-conforming adolescents, regardless of sexual orientation, experience high rates of victimization [9,12,13,14], including high rates of sexual orientation-based and gender-variant-based victimization [15,16] even though the adolescents may be other-sex attracted. However, this work is limited by focusing on adolescents who are gender non-conforming, rather than exploring the consequences of any adolescent being victimized for being perceived as gender-variant.

Examining the association between gender and attraction best highlights the potential for gender-variant-based victimization to impact all adolescents, regardless of same- or other-sex attraction. Gender norms in the United States, and in many places in the world, are heteronormative in that they are based on an assumption that all typical people should want to attract an opposite sex romantic/sexual partner (e.g., males need to behave in ways consistent with masculine stereotypes so that they can attract female romantic/sexual partners) [17]. Thus, being in any way perceived as same-sex attracted is automatically viewed a violation of expected gender norms. However, there are many aspects of a person including self-presentation, interests, and behaviors that can be judged to violate expected gender norms [18,19]. For example, an other-sex attracted female adolescent who plays sports, expresses an interest in being a welder, and prefers to have very short hair would likely be viewed by her peers as violating expected gender norms and would likely be targeted for gender-variant-based victimization, regardless of whether she is attracted to males. If a same-sex attracted male adolescent has the exact same profile of behavior, interests, and self-presentation, his peers would view him as conforming to expected gender norms and would likely not target him for gender-variant-based victimization, as long as they do not find out that he is attracted to males. Therefore, there is nothing about simply being same-sex attracted that would make an adolescent a target for gender-variant-based victimization, but rather it is being publicly identified as being same-sex attracted that alerts his or her peers to a violation of expected gender norms. This assertion is supported by the previous work suggesting that same-sex attracted adolescents are not equally targeted for gender-variation based victimization. Same-sex attracted adolescents who most fit the prescribed feminine gay or masculine lesbian stereotypes report the highest levels of victimization [8,9]. Thus, being attracted to the same-sex may be associated with gender-variant-based victimization based on the self-presentation or self-disclosure of some same-sex attracted people, but it is not the only factor that drives this type of victimization. Thus, there may be a large number of youth who experience gender-variant-based victimization who are not identified in research or reached through interventions that target sexual-minority adolescents.

### The Impact of Gender-Variant-Based Victimization

Gender-variant based victimization has a large impact on adolescents because gender plays a central role in their sense of self and well-being. For example, it has been found that both males and females who feel that they are gender typical are more likely to have a reduced sense of loneliness [18], higher sense of self-worth [18,19], and better overall psychological adjustment [18] compared to those who feel that they are perceived as not expressing their gender appropriately. Importantly, these effects are not based on objective assessments of gender typicality, but rather on how individuals feel like their peers perceive them. Work focused on males has shown that males who are perceived to express more feminine, or gender atypical, traits than their peers report higher levels of victimization, loneliness, and distress [20]. This results in adolescent males’ feeling the need to constantly monitor their self-presentation to defend themselves from being victimized for being perceived as having feminine qualities or not being masculine enough [16]. Thus, managing other peoples’ impressions of ones’ gender expression becomes a constant part of navigating social interactions.

The connection between gender-variant-based victimization and suicidal behaviors and substance use can be understood through the Minority Stress Model [21,22] and the Interpersonal Theory of Suicide [23]. The Minority Stress Model posits that mental health issues and self-medication via substance use can result from the stress people experience when faced with a hostile social environment and social exclusion due to being part of a minority group. While this model has most often been used with traditional minority groups such as sexual minorities [21,22], the key component of this model is that people feel that they are being targeted for an aspect of who they are, and that who they are in someway separates them from the majority group. Thus, an other-sex attracted student who is victimized because some of her peers do not think she acts feminine enough may feel excluded from the majority group because of who she is and how she normally expresses herself. As outlined in a similar theoretical framework connecting the high risk of suicide and substance use among trans people [24], this feeling of social exclusion can result in high levels of stress, which people may try to cope with through substance use. Additionally, this feeling of social exclusion can result in feelings of social isolation, which the Interpersonal Theory of Suicide suggests is associated with being at higher risk for expressing suicidal behaviors [23,24]. Thus, simply experiencing gender-variant-based victimization, a type of victimization directed at people who are seen as not conforming to the majority expectations for gender expression, may result in feelings of social exclusion, which has potential ramifications for any adolescents’ well-being.

### The Current Study

Much of the previous work exploring gender-variant-based victimization has focused on adolescents who were identified as being part of a sexual- or gender-minority group [1]. The current study takes a broader approach to exploring gender-variant-based victimization by focusing on the outcomes of adolescents who report any amount of victimization based on their gender expression, regardless of attraction. To highlight the point that this type of victimization is not just an issue for a subset of youth based on their same-sex attraction, the current study separates out attraction and gender-variation-based victimization as being independently associated with suicidal behavior and substance use. Additionally, the present study compares the association of gender-variant-based victimization with suicidal behavior and substance use between other-sex and same-sex attracted adolescents.

In sum, this study addresses two novel research questions. First, is gender-variant-based victimization related to suicidal behavior and substance use among other-sex attracted adolescents? Second, is the relationship between gender-variant-based victimization and these health-risking behaviors similar between same- and other-sex attracted adolescents? Based on the above review highlighting the negative effects of gender-variant-based victimization, three hypotheses were proposed and examined:

1. Other-sex attracted students who experience gender-variant-based victimization will be more likely to report suicidal behaviors and substance use, compared to other-sex attracted students who do not experience gender-variant-based victimization.
2. Attraction (i.e., same-sex as compared to other-sex) and experiencing gender-variant-based victimization will both independently be associated with higher levels of suicidal behaviors and substance use.
3. The relationship between gender-variant-based victimization and these health-risking behaviors will not differ as a function of attraction (i.e., same-sex as compared to other-sex).

## Method

### Procedures and Ethics Statement

Students completed an anonymous, 30-minute online survey under teacher/staff supervision during school hours in May 2012 as part of a district needs assessment. As part of the district needs assessment process, the school administrators ensured that parents/guardians were: informed of the study, could review the survey, and could opt their child out of participation, in writing, prior to survey administration (fewer than 1% withdrew). As per standard practice in United States School districts, choosing not to opt their child out of participation was taken as parent/guardian consent for their child to participate in the needs assessment. The students themselves were not formally asked to consent to participate in the needs assessment, but they were informed that they could opt out of participating at any point by ceasing to respond to questions and were only required to provide their biological sex and school attended to complete the survey (93% of students participated). The Colorado State University Institutional Review Board approved the procedures used to attain consent and implement the needs assessment, and approved the use of the anonymous data for the purposes of research. This work was deemed as exempt from the requirements of human subject protections by the Colorado State University Institutional Review Board based on the grounds that the research was being conducted in “established or commonly accepted education settings, involving normal education practices, such as a) research on regular and special education strategies, or b) research on the effectiveness of or the comparison among instructional techniques, curricula, or classroom management methods.”

### Measures

#### Gender-variant-based victimization

Based in part on previous work [25], males were asked, “*Do kids ever make fun of you for not being masculine enough/too feminine*?”, and females were asked, “*Do kids ever make fun of you for not being feminine enough/too masculine*?” Participants responded using a Likert-scale (1 = *Never*, 2 = *Sometimes*, 3 = *Often*, 4 = *Always*). A dichotomous variable was created (having never experienced gender-variant-based victimization (0) vs. experiencing gender-variant-based victimization at least sometimes or more often (1).

#### Attraction

Attraction to members of the same- and other-sex was assessed using a single item (*When you are older*, *who might you be interested in dating*? *If you are already dating*, *who are you interested in dating*?), with participants selecting each option that applied (*boys*, *girls*, *or not sure*). A dichotomous variable was created, i.e., participants indicating any same-sex attraction were categorized as same-sex attracted (1), whereas participants indicating only other-sex attraction were categorized as other-sex attracted (0). All participants (*n* = 36), who selected *not sure*, also selected at least one other option allowing for categorization as either same-sex (*n* = 20) or other-sex attracted (*n* = 16).

#### Suicidal ideation

Suicidal ideation during the previous week was assessed using 4 items (α = .87; e.g., *I had thoughts about death*, *I felt that I would kill myself if I knew a way*) [26]. Participants responded to all items using a 4-point scale (1 = *Rarely or none of the time (less than 1 day)*, 2 = *(1–2 days)*, 3 = *(3–4 days)*, 4 = *Most or all of the time (5–7 days)*). A dichotomous variable was created so that all of the participants who reported a 1 on all 4 items were coded as 0, and all participants who reported a 2 or higher on at least 1 of the 4 items were coded as 1.

#### Suicide planning

Suicide planning since the beginning of the school year (past 35–38 weeks) was assessed using 2 items (α = .74; *Since the beginning of the school year have you ever seriously considered attempting suicide (i*.*e*., *tried to kill yourself)*? *and Since the beginning of the school year have you made a plan about how you would attempt suicide*?). Participants responded to each question with either a *Yes* (1) or a *No* (0). A dichotomous composite variable was created by combing the responses to the two questions, i.e., all of the participants who reported a 0 on both items were coded as 0, while all participants who reported a 1 on at least 1 of the 2 items were coded as 1.

#### Suicide attempts

Suicide attempts were assessed using a single item (*Since the beginning of the school year*, *how many times have you actually attempted suicide*?). Participants responded to this item using a 4-point scale (1 = *0 times*, 2 = *1 time*, 3 = *2 or 3 times*, 4 = *4 or more times*). A dichotomous variable was created so that no suicide attempts since the beginning of the school year were indicated with 0; any suicide attempts since the beginning of the school year were indicated with 1.

#### Substance use

Alcohol use (*How often in the past 3 months have you*: *been drunk*?), marijuana use (*How often in the past 3 months have you*: *used marijuana (pot*, *hash*, *reefer)*?), and other drug use (α = .88; *How often in the past 3 months have you*: *used meth*?, and *How often in the past 3 months have you*: *used other drugs*?) were each assessed. Participants responded to each item using an 8-point scale (1 = *Never*, 2 = *Once or twice*, 3 = *About once per month*, 4 = *A few times a month*, 5 = *About once a week*, 6 = *A few times a week*, 7 = *Almost every day*, 8 = *More than once a day*). A dichotomous variable for each type of substance use was created, i.e., never drunk in the past 3 months (0) vs. drunk at any point during the past 3 months (1). Regular cigarette smoking (*How often in the past 3 months have you*: *smoked cigarettes*?) was also assessed using the same 8-point scale. A dichotomous variable was created i.e., did not smoke cigarettes regularly in the past 3 months (0; 1 = *Never*, 2 = *Once or twice*, 3 = *About once per month*, 4 = *A few times a month*, 5 = *About once a week*, 6 = *A few times a week*) vs. did smoke cigarettes regularly in the past 3 months (1; 7 = *Almost every day*, 8 = *More than once a day*).

### Analysis

All models were tested using a logistic regression model in SAS, Version 9.3. For hypothesis 3, we estimated both the multiplicative and additive interaction (i.e., the Relative Excess Risk due to Interaction (RERI)). In logistic regression, the logit coefficient associated with a product of two variables (x*z, for example, bullying based on gender expression and attraction) is an estimate of interaction on a multiplicative scale and a significant interaction denotes that the combined effect of x and z is greater than the *product* of the individual effects of x and z [27]. It has been argued that assessment of the interaction on an additive scale when the outcome is categorical is also useful, particularly if assessment of the interaction has public health relevance [27,28]. Assessment of the additive interaction based on a logistic regression model is commonly done through the calculation of the RERI. A significant RERI denotes that the combined effect of x and z is greater than the *sum* of the individual effects of x and z.

For hypotheses 2 and 3, we report the OR (i.e., exp(*B*)) and 95% CI for all models, a 95% CI that does not contain 1 indicates statistical significance, *p*<.05. For the RERI estimates for hypothesis 3, we also report the 95% CI calculated using the delta method [28], for these, a 95% CI that does not contain 0 indicates statistical significance, *p*<.05.

## Results

### Sociodemograph Characteristics and Preliminary Analyses

Participants were from one school district in the Northeastern United States. A total of 2,944 students started the survey; but only 2,438 (82.8%) completed both the attraction and gender-variant-based victimization questions. Approximately half (50.1%) of the participants were female. The median age was 15 with 51.8% of the students attending one of two middle schools, and the remainder attending the high school. Approximately half were eligible for free/reduced lunch at the time of the survey. Most students, based on school records, were either Black (37%) or Hispanic (35%); about one-quarter of the students (24%) were White, non-Hispanic. Individuals who reported interest in dating a member of the same sex (35 males, 33 females), or both sexes (27 males, 80 females), were categorized as same-sex attracted. This categorization is supported by the similarity between the proportion of both- and same-sex attracted students reporting health-risking behaviors, in conjunction with the dissimilarity between the proportions of both- and other-sex attracted students reporting the same behaviors. Tables 1 and 2 present the sample size, sample proportions, and prevalence of each outcome as a function of attraction. Tables 3 and 4 present the sample size, sample proportions, and prevalence of each outcome as a function of sex for each attraction group.

**Table 1:** Prevalence of suicidal and substance using behaviors as a function of attraction, separating out both-sex attracted students.

	Other-Sex Attracted Only	Same-Sex Attracted Only	Both-Sex Attracted
*n*	2263 (92.8%)	68 (2.8%)	107 (4.4%)
Health-risking behavior			
Suicidal ideation	0.24	0.40	0.44^a^
Suicide attempt	0.07	0.32	0.27^a^
Suicide planning	0.12	0.32	0.45^a^
Alcohol intoxication	0.22	0.07	0.51^a^
Regular cigarette smoking	0.03	0.05	0.19^a^ ^,^ ^b^
Marijuana use	0.16	0.29	0.51^a^ ^,^ ^b^
Other drug use	0.06	0.08	0.25^a^

^a^Denotes a significant difference in the proportion of the health-risking behavior compared with students who are other-sex attracted only.

^b^Denotes a significant difference in the proportion of the health-risking behavior compared with students who are same-sex attracted only.

**Table 2:** Prevalence of suicidal and substance using behaviors as a function of attraction.

	Other-Sex Attracted	Same-Sex Attracted
*n*	2263 (92.8%)	175 (7.2%)
Health-risking behavior		
Suicidal ideation	0.24	0.42^a^
Suicide attempt	0.07	0.29^a^
Suicide planning	0.12	0.40^a^
Alcohol intoxication	0.22	0.50^a^
Regular cigarette smoking	0.03	0.14^a^
Marijuana use	0.16	0.43^a^
Other drug use	0.06	0.24^a^

^a^Denotes a significant difference in the proportion of the health-risking behavior compared with students who are other-sex attracted.

**Table 3:** Prevalence of suicidal and substance using behaviors as a function of sex for same-sex attracted students.

	Males	Females
*n*	62 (35.4%)	113 (64.6%)
Health-risking behavior		
Suicidal ideation	0.44	0.42
Suicide attempt	0.34	0.27
Suicide planning	0.39	0.41
Alcohol intoxication	0.44	0.53
Regular cigarette smoking	0.18	0.12
Marijuana use	0.36	0.47
Other drug use	0.32	0.20

^a^Denotes a significant difference in the proportion of the health-risking behavior compared with students who are males.

**Table 4:** Prevalence of suicidal and substance using behaviors as a function of sex for other-sex attracted students.

	Males	Females
*n*	1154 (51.0%)	1109 (40.0%)
Health-risking behavior		
Suicidal ideation	0.20	0.27^a^
Suicide attempt	0.08	0.06^a^
Suicide planning	0.09	0.16^a^
Alcohol intoxication	0.23	0.20
Regular cigarette smoking	0.05	0.01^a^
Marijuana use	0.19	0.12^a^
Other drug use	0.07	0.04^a^

^a^Denotes a significant difference in the proportion of the health-risking behavior compared with students who are males.

### Overall Descriptives

Table 5 presents the sample size, sample proportions, and prevalence of each outcome as a function of attraction (1 = same-sex, 0 = other-sex) and experience with gender-variant-based victimization (1 = experienced gender-variant-based victimization, 0 = did not experience gender-variant-based victimization). Within each attraction group, we indicate whether the prevalence of each health-risking behavior is significantly different between students who report gender-variant-based victimization and those who do not.

**Table 5:** Prevalence of suicidal and substance using behaviors as a function of attraction and gender-variant-based victimization.

	Other-Sex Attracted Students			Same-Sex Attracted Students		
	Not-Victimized	Victimized		Not-Victimized	Victimized	
*n*	1876 (82.9%)	387 (17.1%)		108 (61.7%)	67 (38.3%)	
Females	941 (84.9%)	168 (15.1%)^a^		74 (65.5%)	39 (34.5%)	
Males	935 (81.0%)	219 (19.0%)^a^		34 (55.8%)	28 (45.2%)	
			Significant			Significant
Health-risking behavior			Difference^b^			Difference^b^
Suicidal ideation	0.20	0.41	*p*<.05	0.34	0.55	*p*<.05
Suicide attempt	0.05	0.16	*p*<.05	0.20	0.43	*p*<.05
Suicide planning	0.10	0.26	*p*<.05	0.31	0.55	*p*<.05
Alcohol intoxication	0.21	0.23	NS	0.46	0.55	NS
Regular cigarette smoking	0.03	0.05	*p*<.05	0.12	0.18	NS
Marijuana use	0.15	0.16	NS	0.40	0.48	NS
Other drug use	0.05	0.08	*p*<.05	0.21	0.29	NS

^a^Denotes a significant difference in the proportion of other-sex attracted students experiencing victimization, *Χ* ^2^ (1, n = 2263) = 5.85, *p* <.05.

^b^Denotes a significant difference in the prevalence of the health-risking behavior by victimization status within attraction type, NS = not significant.

Overall, 18.6 percent of students reported experiencing gender-variant-based victimization. A larger proportion of same-sex attracted students (38.3%) reported experiencing gender-variant-based victimization than other-sex attracted students (17.1%), *Χ* ^2^ (1, n = 2438) = 48.11, *p* < .05. Additionally, a larger proportion of males (20.3%) reported experiencing gender-variant-based victimization than females (16.9%), *Χ* ^2^ (1, n = 2438) = 4.58, *p* < .05. However, there was not a significant difference between middle school (18.1%) and high school (19.2%) students in the proportion of students who reported experiencing gender-variant-based victimization, *Χ* ^2^ (1, n = 2438) = .44, n.s.

### Hypothesis Tests

Congruent with hypothesis 1, other-sex attracted students who experienced gender-variant-based victimization were more likely than other-sex attracted students who did not experience gender-variant-based victimization to report suicidal ideation in the past week, suicidal attempts since the beginning of the school year, suicide planning since the beginning of the school year, regular cigarette smoking in the past 3 months, and other drug use in the past 3 month. However, gender-variant-based victimization was not associated with alcohol intoxication and marijuana use in the past 3 months among other-sex attracted youths.

A similar pattern is observed for same-sex attracted youths in terms of suicide-related behaviors. That is, same-sex attracted youths who reported gender-variant-based victimization were more likely report suicidal ideation in the past week, and both suicidal attempts and suicide planning since the beginning of the school year. The association between substance use and gender-variant-based victimization is in the expected direction, although it doesn’t meet traditional criteria for statistical significance.

To test hypothesis 2, we regressed each of the outcomes on attraction and experiencing gender-variant-based victimization to determine if each is uniquely associated with self-reports of the health-risking behaviors. These results are presented in Table 6, Model 1. Controlling for attraction, students who experienced gender-variant-based victimization were more likely than students who did not experienced gender-variant-based victimization to report suicidal ideation in the past week, suicidal attempts since the beginning of the school year, suicide planning since the beginning of the school year, and regular cigarette smoking and other drug use in the past 3 months. However, gender-variant-based victimization was not associated with alcohol intoxication in the past 3 months or marijuana use in the past 3 months. Controlling for experiencing gender-variant-based victimization, same-sex attracted students were more likely than their other-sex attracted peers to report all of the suicide-related behaviors and all forms of substance use. These findings suggest that gender-variant-based victimization and same-sex attraction each uniquely associated with health-risking behaviors. These results provide general support for hypothesis 2.

**Table 6:** Results of logistic regression models to test hypothesis 2 and 3.

	Model 1			Model 2					
		95% CI exp(*B*)			95% CI exp(*B*)			95% CI RERI	
	*exp(B)*	*-*	*+*	*exp(B)*	*-*	*+*	*RERI*	*-*	*+*
Health-risking behavior									
Suicidal ideation									
Intercept	0.25	-0.23	0.28	0.25	-0.22	0.28			
Attraction	1.95	-1.40	2.70	2.08	-1.38	3.14			
Victimization	2.75	-2.22	3.42	2.81	-2.23	3.55			
Attraction*Victimization				0.84	-0.43	1.64	1.03	-1.54	3.60
Suicide attempt									
Intercept	0.05	-0.04	0.06	0.05	-0.04	0.06			
Attraction	4.52	-3.09	6.62	5.05	-3.02	8.44			
Victimization	3.60	-2.64	4.89	3.78	-2.68	5.33			
Attraction*Victimization				0.79	-0.37	1.68	7.24	-0.60	15.07
Suicide planning									
Intercept	0.11	-0.09	0.13	0.11	-0.09	0.12			
Attraction	3.91	-2.78	5.50	4.17	-2.69	6.46			
Victimization	3.17	-2.46	4.08	3.25	-2.47	4.28			
Attraction*Victimization				0.85	-0.43	1.70	5.11	-0.78	11.01
Alcohol intoxication									
Intercept	0.27	-0.24	0.30	0.27	-0.24	0.30			
Attraction	3.49	-2.54	4.79	3.16	-2.13	4.69			
Victimization	1.13	-0.89	1.43	1.08	-0.83	1.40			
Attraction*Victimization				1.33	-0.68	2.58	1.28	-1.22	3.78
Regular cigarette smoking									
Intercept	0.03	-0.02	0.03	0.03	-0.02	0.03			
Attraction	4.89	-2.96	8.08	5.21	-2.73	9.94			
Victimization	1.77	-1.11	2.83	1.86	-1.07	3.23			
Attraction*Victimization				0.86	-0.31	2.37	2.24	-3.84	8.32
Marijuana use									
Intercept	0.18	-0.16	0.20	0.18	-0.16	0.21			
Attraction	4.02	-2.90	5.56	3.62	-2.41	5.43			
Victimization	1.08	-0.83	1.42	1.02	-0.76	1.38			
Attraction*Victimization				1.35	-0.68	2.68	1.36	-1.43	4.15
Other drug use									
Intercept	0.06	-0.05	0.07	0.06	-0.04	0.07			
Attraction	4.78	-3.21	7.11	4.96	-3.00	8.22			
Victimization	1.59	-1.11	2.28	1.63	-1.08	2.47			
Attraction*Victimization				0.90	-0.40	2.05	1.73	-2.84	6.30

Notes: exp(*B*) = exponentiated estimate, CI = confidence interval, RERI = relative excess risk due to interaction.

Attraction is coded 1 if same-sex attracted, 0 if other-sex attracted. Victimization is coded 1 if victimized, 0 if not victimized.

95% CI exp(*B*) is statistically significant when the CI doesn't include 1.

95% CI RERI is statistically significant when the CI doesn't include 0.

To test hypothesis 3, we enhanced the models specified to test hypothesis 2 by adding an interaction term between victimization and attraction to determine if the effect of experiencing gender-variant-based victimization differed by attraction. The results are presented in Table 6, Model 2. Across all of the outcomes, none of the interaction terms were statistically significant, and this holds for both the multiplicative and additive interaction terms. These findings suggest that the effect of experiencing gender-variant-based victimization does not significantly differ as a function of attraction, providing support for hypothesis 3.

## Discussion

This study set out to examine the association between gender-variant-based victimization and negative outcomes (i.e., suicidal behavior, substance use) in other-sex attracted adolescents, and to highlight gender-variant-based victimization and attraction as uniquely associated with suicidal behavior and substance use for adolescents. As hypothesized, it was found that other-sex attracted adolescents who experienced gender-variant-based victimization were more likely to report suicidal behaviors than other-sex attracted adolescents who did not experience this type of victimization.

Additionally, as predicted, same-sex attraction and experiencing gender-variant-based victimization were both found to be associated with suicidal behaviors, and the effect of gender-variant-based victimization was not found to be modified by attraction. However, our hypotheses were only partially supported for the substance use measures. Other-sex attracted adolescents who experienced gender-variant-based victimization were more likely to report regular cigarette smoking and other drug use than those who did not experience this type of victimization. Controlling for attraction, gender-variant-based victimization was associated with self-reports of regular cigarette smoking and other drug use. However, the association between experiencing gender-variant-based victimization and suicidal behavior and substance use does not vary as a function of attraction. Thus, overall, our findings suggest that for both other- and same-sex attracted adolescents experiencing gender-variant-based victimization is associated with an increased likelihood of reporting suicidal behaviors, and some types of substance use, especially for other-sex attracted adolescents.

The results of the present study are consistent with previous findings suggesting that expressing gender atypical characteristics during adolescence [8,9], or experiencing gender-variant-based victimization [8,25,29], are related to lower levels of psychological well-being, regardless of being same- or other-sex attracted. Extending from these studies, the present study highlights the association between gender-variant-based victimization and suicidal behavior and substance use in other-sex attracted adolescents. Thus, this study helps to move the present literature beyond almost exclusively focusing on negative outcomes associated with gender-variant-based victimization in specific minority groups (i.e. sexual- and gender-minorities), and provides an opportunity to discuss the broader issue of gender policing in adolescents.

### Implications

The current work has potential implications for interventions addressing types of bullying that have gender-variant-based victimization components. For example, over the last several years, homophobic bullying has been identified as a key risk factor for suicidal behavior, substance use, and other negative health outcomes in adolescents [3,29,30]. This has resulted in research mostly focusing on identification as lesbian, gay, bisexual (LGB), questioning, or being same-sex attracted as risk factors for negative health outcomes [3,4], or for experiencing victimization [31,32]. However, this work does little to address the motives behind homophobic bullying and the forms of victimization that fall under its broad umbrella. For example, same-sex attracted adolescents may be target for victimization solely because of their sexual orientation, but often times they are targeted for victimization because their identity as being same-sex attracted is not viewed by their peers as being consistent with traditional gender norms [15,16]. In these instances, victimization is gender-variant-based and used as a form of peer gender policing. Thus, same-sex attracted adolescents who experiences homophobic bullying may be targeted exclusively for sexual orientation-based victimization, but may also targeted for gender-variant-based victimization.

While exploring gender-variant-based victimization specifically as a component of homophobic bullying is beyond the scope of this the current work, others have noted that homophobic bullying is made up of both sexual orientation-based victimization and gender-variant-based victimization [15,16,33,34]. Even though these two types of victimization are very closely linked, and both likely disproportionately impact sexual- and gender-minority adolescents, their underlying motives have implications for who and how interventions are targeted. For example, if homophobic bullying is indeed mostly sexual orientation-based, then focusing on identifying and supporting sexual-minority students and promoting acceptance of the diversity in sexual orientation is likely the best approach.

Yet, if homophobic bullying is mostly driven by policing gender through the use of gender-variant-based victimization, the issues becomes much broader because any adolescents, regardless of sexual orientation, may become targets for homophobic bullying based on the way they express their gender. Indeed, some previous work demonstrates the effects of homophobic bullying on the psychological and social wellbeing of adolescents, regardless of sexual orientation [28]. The current work does not capture whether the gender-variant-based victimization that other-sex students experienced was homophobic in nature, but it does demonstrate the broad impact that gender-variant-based victimization can have on both other- and same-sex adolescents.

This study also identifies gender-variant-based victimization as being uniquely associated with health-risking behavior, especially suicidal behavior. Further work is needed to understand the specific mechanisms that underlie this association. However, if this relationship is found to be explained by feelings of social exclusion created by this type of victimization as previous work suggests [24], then our study would support creating broad-based interventions that focus on general acceptance of gender diversity. Focusing on promoting acceptance for individual differences in gender expression is a qualitatively different approach than targeting interventions to sexual- and gender- minority adolescents who are stereotyped as being gender atypical and experiencing this type of victimization.

### Strengths & Limitations

This work has several strengths. First, this study utilizes a large, diverse sample of both middle school and high school students in the Eastern United States. Given the relatively progressive nature of this region of the country, the presence of gender-variant-based victimization and its association with negative outcomes as serious as suicidal behavior and substance use is notable. Additionally, this work is strengthened by its investigation of gender-variant-based victimization across the entire sample of adolescents. Most previous work addressing gender-variant-based victimization has focused on subsets of the adolescent population that are stereotyped as expressing gender-atypical traits (i.e. lesbian, gay, and bisexual adolescents) or who are identified as being gender-atypical (e.g., transgender or gender nonconforming). The current work, instead, asked all students if they had experienced victimization based on their gender expression.

Another key strength of this work is that it separates the effects of gender-variant-based victimization from the effects of attraction in their association with suicidal behavior and substance use. This helps to highlight the similar negative effects experienced by both other-sex and same-sex attracted youth who are targeted for gender-variant-based victimization. Additionally, this analysis demonstrates that gender-variant-based victimization is not something that is universally experienced by same-sex attracted youth, helping to account for some of the heterogeneity among same-sex attracted adolescents in their experiences with and outcomes related to victimization.

One of the primary limitations to the current work is that the investigators did not ask participants to describe the nature of the victimization that they identified as being based on their gender expression. Thus, there is no way to know exactly what types of behaviors and language were identified as being gender-variant-based victimization in this work. However, for this study, the researchers were intentional about providing a broad question that was devoid of any terms related to sexuality, attraction, or other identities so as to better account for the experiences of all students, not just students who are same-sex attracted or identify as transgender or gender nonconforming.

This study is also limited in that the sample was only obtained from one school district, but due to the large sample size and the diversity of the students who participated, the effects of this limitation on the outcome of this study are believed to be minimal. Additionally, despite our large sample size, our study may have still been underpowered when it comes to investigating the association between gender-variant-based victimization and substance use for same-sex attracted adolescents. This is evidenced by results for the substance use outcomes trending in the predicted direction. It should also be noted that our study may have been underpowered for detecting an interaction between attraction and experiencing gender-variant-based victimization. The statistical analyses conducted did not find a multiplicative or an additive interaction for any of the health-risking behaviors assessed, but it is not possible for the current work rule out the existence of an interaction between these two constructs. However this study does suggest that the effects of any potential relationships between an interaction and the assessed health-risking behaviors are weaker than the effects of attraction and gender-variant-based victimization in of themselves. Future studies with larger, multi-school district samples will be needed to help fully tease apart these relationships and provide more clarity with regard to the nuances of the lesser effects.

This work is also limited by the way that same-sex attracted adolescents were identified and categorized. The actual identification item itself focused on future dating intentions, and therefore it does not provide a direct assessment of self-identification as a sexual minority, limiting this work in the broader literature on sexual minority adolescents. However, this single item provided a developmentally appropriate way to capture attraction from adolescents spanning both middle school and high school.

Additionally, adolescents who expressed attraction to both males and females were coarsely grouped with adolescents who expressed exclusive same-sex attraction to create a single comparison group. While this grouping combines two distinctly different groups, this decision was based on preliminary analyses demonstrating exclusively same-sex attracted adolescents and adolescents expressing both same- and other-sex attracted were similar to one another, and similarly different from exclusively other-sex attracted adolescents, on all of the variables assessed in this study. Thus, this decision did not alter the overall findings of the study. A similar potential limitation comes from grouping both males and females together within each of the attraction groups; however, this decision was also based on preliminary analyses indicating that the general pattern of results held true for both males and females. Therefore, combining males and females did not alter the overall findings of the study. In the end, these findings are based on a large, diverse sample, and show strong relationships, despite only focusing on suicidal behavior and substance use over a relatively short amount of time, supporting the assertion that these findings are not just due to chance or particularly unique conditions.

### Future Research

Future work may benefit from following-up with a subset of participants in focus groups to better understand the specific actions and language that they believe constitutes gender-variant-based victimization. The current study identifies gender-variant-based victimization as a factor associated with health-risking behavior through the use of a question asking students whether they have been made fun of for how they express their gender (i.e., *Do kids ever make fun of you for not being feminine enough/too masculine*?), but this simple item does not provide any information about why the adolescents perceived the victimization as being based on their gender expression. Collecting additional information through focus groups could help to identify the types of behaviors and language that are associated with this form of victimization. Additionally, this work may benefit from being replicated across multiple school districts in different parts of the country to ensure the findings are not just an artifact of the specific culture and experiences of students in this particular school district. Finally, it is recommended that interventions developed to reduce bullying and its negative outcomes are designed with an understanding that gender-variant-based victimization is an issue for all students, and not just specific target populations. Thus, adolescents might benefit from creating a school culture that is accepting of the diversity of all students’ expressions of gender, regardless of their actual or perceived sexual orientation or gender identity. However, this is an empirical question that will need to be addressed in further work.

## Conclusions

The current work uniquely contributes a link between gender-variant-based victimization and suicidal behavior and substance use. For all adolescents (both other- and same-sex attracted), gender-variant-based victimization was associated with a higher odds of suicidal thoughts and behaviors. In addition, for other-sex attracted adolescents, gender-variant-based victimization was associated with increased odds of smoking cigarettes regularly and using other drugs. These findings suggest that gender-variant-based victimization has potentially serious implications for the psychological wellbeing and substance use of other-sex attracted adolescents, not just same-sex attracted adolescents. Thus, gender-variant-based victimization has implications for all adolescents, not just a subset of adolescents that are typically considered to be at risk for this this type of victimization based on their identity. Taken together, the findings of this study highlight the need to better understand the influence that gender-variant-based victimization has on all adolescents. From a prevention and intervention standpoint, this work suggests that additional attention may need to be paid to all adolescents who do not conform to gender norms or who are bullied based on their gender expression. Additionally, educators, researchers, and providers may need to focus on the broader issue of creating acceptance for a diverse array of gender expressions, instead of focusing on specific minority groups, in order to reduce negative outcomes such as suicidal behaviors and substance use. This may be especially true when addressing homophobic or gender-based bullying because much of this victimization stems from the policing of gender expression through the use of gender-variant-based victimization.

## Supporting Information

S1 File This the Dataset File. This file contains all of the data for the analyses conducted in this study.
(XLS)
