# Correction: Beyond Same-Sex Attraction: Gender-Variant-Based Victimization Is Associated with Suicidal Behavior and Substance Use for Other-Sex Attracted Adolescents

**DOI:** 10.1371/journal.pone.0139532

**Published:** 2015-09-25

**Authors:** Michael Ioerger, Kimberly L. Henry, Peter Y. Chen, Konstantin P. Cigularov, Rocco G. Tomazic

There are errors in Table 6, “Results of logistic regression models to test hypothesis 2 and 3.” Please see the corrected Table 6 here.

**Table 6:** Results of logistic regression models to test hypothesis 2 and 3.

	Model 1			Model 2					
		95% CI exp(*Β*)			95% CI exp(*Β*)			95% CI RERI	
	*exp(Β)*	*-*	*±*	*exp(Β)*	*-*	*±*	*RERI*	*-*	*±*
Health-risking behavior									
Suicidal ideation									
Intercept	0.25	0.23	0.28	0.25	0.22	0.28			
Attraction	1.95	1.40	2.70	2.08	1.38	3.14			
Victimization	2.75	2.22	3.42	2.81	2.23	3.55			
Attraction*Victimization				0.84	0.43	1.64	1.03	-1.54	3.60
Suicide attempt									
Intercept	0.05	0.04	0.06	0.05	0.04	0.06			
Attraction	4.52	3.09	6.62	5.05	3.02	8.44			
Victimization	3.60	2.64	4.89	3.78	2.68	5.33			
Attraction*Victimization				0.79	0.37	1.68	7.24	-0.60	15.07
Suicide planning									
Intercept	0.11	0.09	0.13	0.11	0.09	0.12			
Attraction	3.91	2.78	5.50	4.17	2.69	6.46			
Victimization	3.17	2.46	4.08	3.25	2.47	4.28			
Attraction*Victimization				0.85	0.43	1.70	5.11	-0.78	11.01
Alcohol intoxication									
Intercept	0.27	0.24	0.30	0.27	0.24	0.30			
Attraction	3.49	2.54	4.79	3.16	2.13	4.69			
Victimization	1.13	0.89	1.43	1.08	0.83	1.40			
Attraction*Victimization				1.33	0.68	2.58	1.28	-1.22	3.78
Regular cigarette smoking									
Intercept	0.03	0.02	0.03	0.03	0.02	0.03			
Attraction	4.89	2.96	8.08	5.21	2.73	9.94			
Victimization	1.77	1.11	2.83	1.86	1.07	3.23			
Attraction*Victimization				0.86	0.31	2.37	2.24	-3.84	8.32
Marijuana use									
Intercept	0.18	0.16	0.20	0.18	0.16	0.21			
Attraction	4.02	2.90	5.56	3.62	2.41	5.43			
Victimization	1.08	0.83	1.42	1.02	0.76	1.38			
Attraction*Victimization				1.35	0.68	2.68	1.36	-1.43	4.15
Other drug use									
Intercept	0.06	0.05	0.07	0.06	0.04	0.07			
Attraction	4.78	3.21	7.11	4.96	3.00	8.22			
Victimization	1.59	1.11	2.28	1.63	1.08	2.47			
Attraction*Victimization				-0.90	0.40	2.05	-1.73	-2.84	6.30

Notes: exp(*Β*) = exponentiated estimate, CI = confidence interval, RERI = relative excess risk due to interaction.

Attraction is coded 1 if same-sex attracted, 0 if other-sex attracted. Victimization is coded 1 if victimized, 0 if not victimized.

95% CI exp(*Β*) is statistically significant when the CI doesn't include 1.

95% CI RERI is statistically significant when the CI doesn't include 0.

In addition, the authors have provided an updated S1 File, as the original file includes data for variables unrelated to this article. Please view the corrected file below.

S1 File This is the Dataset File. This file contains all of the data for the analyses conducted in this study.
(XLS)
